# Ensembl 2020

**DOI:** 10.1093/nar/gkz966

**Published:** 2019-11-06

**Authors:** Andrew D Yates, Premanand Achuthan, Wasiu Akanni, James Allen, Jamie Allen, Jorge Alvarez-Jarreta, M Ridwan Amode, Irina M Armean, Andrey G Azov, Ruth Bennett, Jyothish Bhai, Konstantinos Billis, Sanjay Boddu, José Carlos Marugán, Carla Cummins, Claire Davidson, Kamalkumar Dodiya, Reham Fatima, Astrid Gall, Carlos Garcia Giron, Laurent Gil, Tiago Grego, Leanne Haggerty, Erin Haskell, Thibaut Hourlier, Osagie G Izuogu, Sophie H Janacek, Thomas Juettemann, Mike Kay, Ilias Lavidas, Tuan Le, Diana Lemos, Jose Gonzalez Martinez, Thomas Maurel, Mark McDowall, Aoife McMahon, Shamika Mohanan, Benjamin Moore, Michael Nuhn, Denye N Oheh, Anne Parker, Andrew Parton, Mateus Patricio, Manoj Pandian Sakthivel, Ahamed Imran Abdul Salam, Bianca M Schmitt, Helen Schuilenburg, Dan Sheppard, Mira Sycheva, Marek Szuba, Kieron Taylor, Anja Thormann, Glen Threadgold, Alessandro Vullo, Brandon Walts, Andrea Winterbottom, Amonida Zadissa, Marc Chakiachvili, Bethany Flint, Adam Frankish, Sarah E Hunt, Garth IIsley, Myrto Kostadima, Nick Langridge, Jane E Loveland, Fergal J Martin, Joannella Morales, Jonathan M Mudge, Matthieu Muffato, Emily Perry, Magali Ruffier, Stephen J Trevanion, Fiona Cunningham, Kevin L Howe, Daniel R Zerbino, Paul Flicek

**Affiliations:** European Molecular Biology Laboratory, European Bioinformatics Institute, Wellcome Genome Campus, Hinxton, Cambridge CB10 1SD, UK

## Abstract

The Ensembl (https://www.ensembl.org) is a system for generating and distributing genome annotation such as genes, variation, regulation and comparative genomics across the vertebrate subphylum and key model organisms. The Ensembl annotation pipeline is capable of integrating experimental and reference data from multiple providers into a single integrated resource. Here, we present 94 newly annotated and re-annotated genomes, bringing the total number of genomes offered by Ensembl to 227. This represents the single largest expansion of the resource since its inception. We also detail our continued efforts to improve human annotation, developments in our epigenome analysis and display, a new tool for imputing causal genes from genome-wide association studies and visualisation of variation within a 3D protein model. Finally, we present information on our new website. Both software and data are made available without restriction via our website, online tools platform and programmatic interfaces (available under an Apache 2.0 license) and data updates made available four times a year.

## INTRODUCTION

Ensembl (https://www.ensembl.org) is a genome annotation and dissemination platform capable of integrating and summarising experimental data against reference genomes and works towards three goals: annotating the vertebrate subphylum, enabling genomic interpretation, and supporting researcher driven analysis. We work extensively with publicly available datasets submitted to archives such as INSDC (1), dbSNP (2), Roadmap Epigenomics (3) and GTEx (4). Our analysis methods are capable of annotating genes and transcripts (5), performing comparative genomics (6), integrating variation data sets from diverse resources (7) and annotating regulatory activity (8) to create a comprehensive and consistent baseline of reference annotation. These data can be accessed via our website, Perl application programming interface (API) (9), RESTful API (10) and tools. We release both data and code four times a year and work alongside our companion resource Ensembl Genomes (11) to deliver a pair of comprehensive services spanning the tree of life.

The past year has seen biodiversity sequencing projects, such as the Vertebrate Genomes Project (VGP, https://vertebrategenomesproject.org/) and Darwin Tree of Life Project (DToL), deliver high-quality genome assemblies. These projects are working as part of the Earth BioGenome Project (12) and come with the promise of delivering assemblies and annotation for all known species for less than the total cost of the Human Genome Project (13). These biodiversity projects are the driving factor behind our developments to scale automated gene annotation (14). Since Ensembl release 94 (October 2018), we have annotated 74 new genomes and re-annotated 20 existing key genomes. Our re-annotation of the pig reference genome, subsequent annotation of 11 pig breeds and extensive comparative genomics analysis represents the most comprehensive and consistent annotation available for swine.

In addition to expanding our annotation of vertebrates, we provide extensive resources to support genome and variant interpretation. Our regulatory build summarises epigenetic activity in 197 epigenomes across human and mouse. We have also developed a new analysis method to impute candidate causal genes from genome-wide association studies (GWAS) using expression quantitative trait loci (eQTLs). Our popular Ensembl Variant Effect Predictor (VEP) (15) can access this new tool and can display genomic variants in the context of 3D protein structure. As part of our continued efforts to support clinical research and data sharing, we have launched two new projects. Matched Annotation from NCBI and EMBL-EBI (MANE) aims to improve concordance between the two major human genome annotation groups: Ensembl/GENCODE (16) and NCBI RefSeq (17). Ensembl Transcript Archive (Tark) helps to track transcripts across different annotation sources and genome builds and identify differences ensuring calculated consequences and acquired knowledge may be propagated between annotation releases.

As data volumes continue to increase, so must our infrastructure develop to meet researcher demands. We have begun a redesign of our website into a responsive application capable of integrating annotation from across the domain of life. As biodiversity projects continue to gather momentum, these developments ensure Ensembl remains a world-class resource for genome researchers.

## PAN-VERTEBRATE GENOME ANNOTATION

### Expanding annotation across the vertebrate domain of life

The continued democratization of sequencing technologies, improvements in assembly methods and large biodiversity projects has made a plethora of high-quality assemblies available for annotation and integration into Ensembl. Our annotation methods, detailed later, continue to use a combination of high-quality evidence such as RNA-seq transcriptomics alongside projection of annotation from a related species and the alignment of UniProt (18) vertebrate proteins. All of our comparative resources have been updated including whole genome alignments (WGAs), orthology and conservation analysis.

As of Ensembl release 98, we have annotated 27 new bird and reptile assemblies. This includes three species of kiwi (little spotted kiwi, great spotted kiwi and Okarito brown kiwi), the great tit and blue tit, the mainland tiger snake, the Australian saltwater crocodile and the tuatara lizard. Eight new rodents have been annotated including the Arctic and Daurian ground squirrels, the alpine marmot and the American beaver. We have also annotated the latest Chinese hamster ovary (CHO) cell line assembly CriGri-PICR (GCA_003668045.1) bringing the total supported CHO assemblies to three. GRCm38 has received three annotation updates (M20–M22) with Ensembl/GENCODE M21 representing the first full manual annotation pass in mouse (released April 2019). Twelve new fish assemblies have been annotated including the first electric fish in the electric eel assembly, where its annotation will prove invaluable in deciphering how electric organs have developed.

Our coverage of farmed and companion animals continues to grow. We have annotated the new reference cow assembly (ARS-UCD1.2) (19), the American bison alongside both maternal and paternal haplotypes for the *Bos indicus* × *Bos taurus* hybrid cattle assemblies alongside WGAs against the reference cow assembly. In swine, we have annotated the U.S. Meat Animal Research Center's PacBio cross breed assembly (Landrace–Duroc–Yorkshire), 11 pig breeds using 8 million filtered long-reads and reannotation of Sscrofa11.1 (20). All strains have been aligned back to the reference pig assembly alongside analyses centred on the *Sus* genus: multiple WGA, orthologies and gene trees. Other updated farmed and companion animals include chicken, duck, horse, dog and cat. In dog, our annotated gene count decreased by 5% to 30 951, whilst transcripts increased by 56% to 60 994, due to the availability of transcriptomic data and annotation analysis improvements.

The aforementioned species expansion has necessitated a number of analysis enhancements. We have adopted Minimap2 (21) as our long read mapper and in our tests it was capable of aligning TTN’s longest transcript ENST00000589042.5 (109 224 bp long with 363 exons) back to GRCh38 without error. Splice site errors are now corrected via a majority rule consensus approach, using nearby intron–exon boundaries and weighted introns from other sources including short read data and introns found via protein homology annotation projection. Finally, we have refined our homology-based method of annotating of well characterized small non-coding RNA (sncRNA). We have worked with NCBI RefSeq to adopt a common set of clade-specific search parameters when retrieving conserved RNA family sequences from Rfam version 14.0 (22).

As a result of our new and updated gene annotation, we have updated our microarray probe mappings against a number of species including human (GRCh37 and GRCh38), mouse, fruit fly, cow, chicken, and dog. Microarray probe mappings have been added for 10 species including common mallard, Chinese hamster (CriGri-PICR), sheepshead minnow, horse and mummichog.

### Improving comparative analysis across the vertebrate domain

The Ensembl project provides a suite of comparative analysis methods to support genome annotation and interpretation. Our multiple sequence high-quality reference free WGA aligner Enredo-Pecan-Ortheus (EPO) now handles genomes previously deemed too fragmented to include. EPO’s low coverage method can now select one of multiple candidate reference genomes based on genetic distance instead of using a single reference for all. For example, our analysis method previously used GRCh38 as the reference genome for wild yak and American bison resulting in 45% and 49% alignment coverage respectively. Our new method now chooses ARS-UCD1.2 as the reference genome, increasing alignment coverage of wild yak to 84% and American bison to 94%. Our orthology analysis now performs d*N*/d*S* on all quality control (QC) passing gene pairs, increasing d*N*/d*S* coverage by 60% to a total of 23.7 million gene pairs.

### Improving reference human annotation

The past year has seen an extensive collaboration with the NCBI RefSeq group to improve the concordance of transcript annotation between the Ensembl/GENCODE and RefSeq annotation sets for human. The MANE project aims to agree on a matched representative transcript for every human protein coding gene, called MANE Select. Each Ensembl/GENCODE transcript tagged as part of MANE Select has a corresponding RefSeq accession with an identical splicing pattern and identical untranslated regions. MANE Select allows a researcher to exchange data and translate coordinates between the two annotation sets. The first release of MANE Select (v0.5) in Ensembl release 96 (April 2019) covered 53% of human protein coding genes. MANE Select v0.6 (Ensembl release 98) increased our coverage to 67%. MANE Select tagged transcripts are visible on our transcript summary tables, integrated into our data mining platform BioMart (23) and available from Ensembl VEP.

### Deeper annotation of genome regulation

The Ensembl regulatory build offers a consistent set of regulatory elements in human and mouse across a diverse range of epigenomes, i.e. the epigenomic profiles of cell types, cell lines or tissues, and annotates six element types: CTCF binding, enhancers, promoter flanking regions, promoters, transcription factor binding sites of unknown provenance and regions of open chromatin. We now cover 118 human epigenomes cataloguing 613 944 elements covering 21% of GRCh38 from over 8TB of experimental data from Roadmap Epigenomics, ENCODE (24) and BLUEPRINT (25). All data sets are available via the International Human Epigenome Consortium (IHEC) (26).

**Figure 1.**
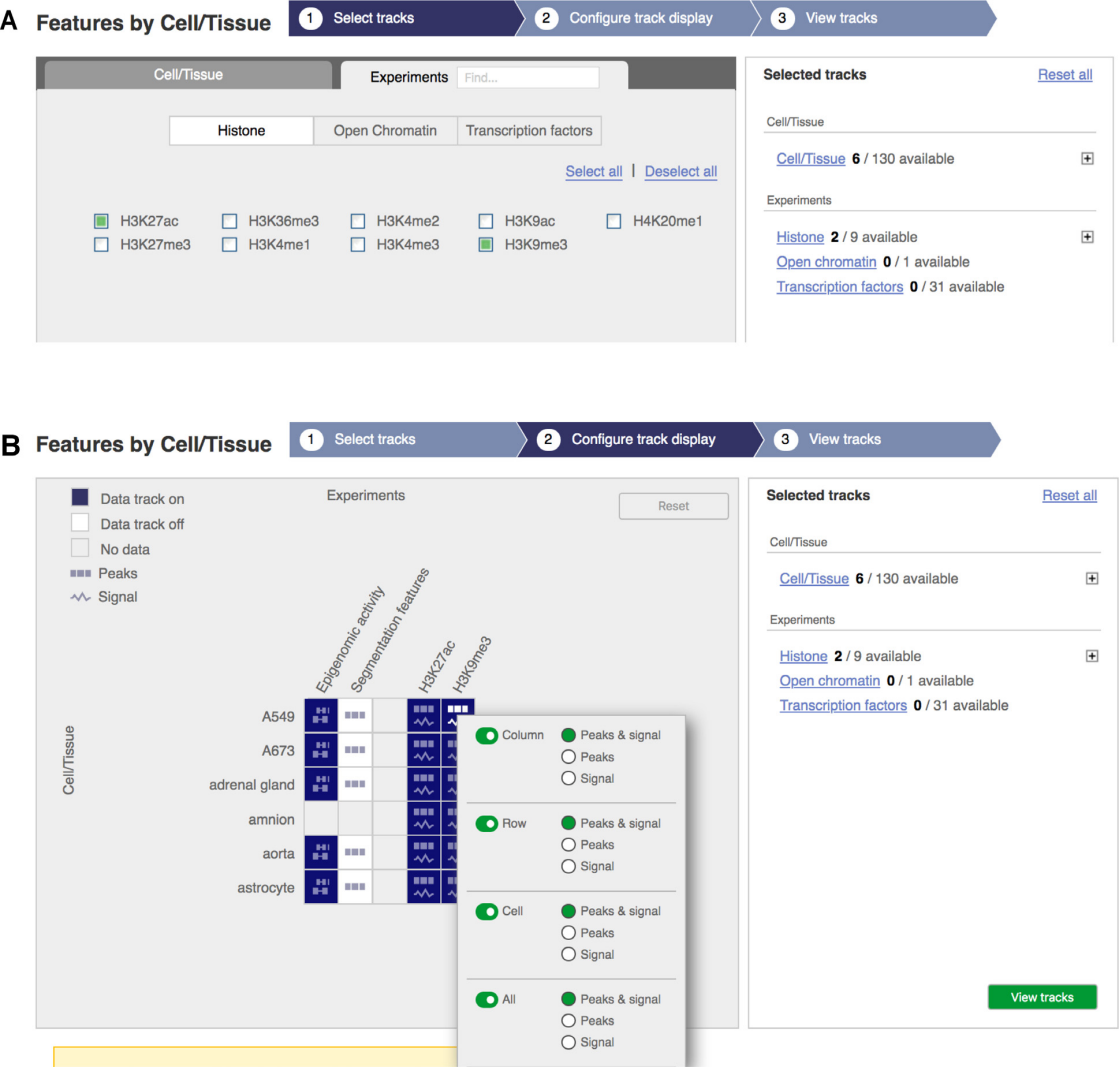
The new epigenome track selection interface, providing a way to find experimental evidence of interest based on cell/tissue and experiment type and to turn those tracks of evidence on in the genome browser. (**A**) Experiments are grouped by their target of interest i.e. histone modifications (e.g. H3K27ac), open chromatin (e.g. DNaseI hypersensitivity) or transcription factors (e.g. CTCF). Cells and tissues of interest can be selected by clicking on the Cell/Tissue tab. This interface has filtered available tracks by six cells/tissues and two histone modifications. (**B**) A matrix view of all available tracks of evidence based on the previously selected cells/tissues and experiments. Tracks of evidence, peaks and signals, can be turned on and off based on the cell/tissue (rows), experiment (columns) or individual cells.

Increasing the volume of our data has necessitated two changes to our systems. Firstly, we have implemented a tighter QC process with extensive statistics produced at multiple stages within the regulatory build. This includes statistics on raw read files, QC reports generated by FastQC (https://www.bioinformatics.babraham.ac.uk/projects/fastqc/), CHANCE (27) and FRIP (28), and finally statistics on the results of peak calling and segmentation. Secondly, we have redesigned our epigenome explorer and track selection interface, as can be seen in Figure 1, and has been through extensive design and user testing to ensure usability in the face of ever-increasing numbers of publicly available epigenomes. Finally, we our miRNA target datasets for human and mouse are imported from the TarBase v8.0 resource (29).

### Variation annotation

We have further enhanced our resources to facilitate improved understanding of genomic variation. We continue to integrate variant data from NCBI dbSNP and the European Variation Archive (EVA) (https://www.ebi.ac.uk/eva/) alongside population frequency data from resources including the Genome Aggregation Database (gnomAD) (30). This year we incorporated dbSNP version 152, which employs new normalization and data distribution methods. Variant sets submitted to EVA can now be seamlessly integrated within our genome browser as demonstrated with vervet monkey (EVA project PRJEB22989). We have improved our annotation of non-coding variants and made Combined Annotation-Dependent Depletion (CADD; (31)) scores available for human and Genomic Evolutionary Rate Profiling (GERP) (32) scores available for all mammals. These data provide an indication as to how tolerant a locus is to change.

## ENABLING GENOME INTERPRETATION

### Causal gene imputation from GWAS studies

Ensembl release 98 saw the release of a Post-GWAS Analysis Pipeline (https://github.com/Ensembl/postgap) to help genome-wide association studies (GWAS) impute causal genes. GWAS summary statistics (beta effect sizes and *P*-values) are fine-mapped taking into account linkage disequilibrium and co-localization analysis with any available eQTL data sets. The analysis returns a list of fine-mapped variants with posterior probabilities, colocalized genes and enriched pathways. Currently, the service performs this analysis against GTEx v6p gene-SNP correlations. In the near future, EMBL-EBI’s eQTL Catalogue (http://www.ebi.ac.uk/eqtl) will provide additional eQTL datasets to analyse against and will be integrated into our service.

### Improving variant interpretation and reporting

We have continued to enhance Ensembl VEP with a number of extensions. Citation information from the Genomenon Mastermind database and tissue-specific transcription factor motifs from the FunMotifs resource (33) can be optionally reported. Additionally, results from our post GWAS analysis pipeline are available through VEP. We have also increased the range of options available for the analysis of structural variants and created a plugin to report information from overlapping structural variant sets, such as gnomAD’s population frequency resource (34). Our G2P plugin (35) has been updated to accept gene panels in the PanelApp format. To aid data integration from disparate resources, we expanded the range of HGVS nomenclature understood by our tools and support the new NCBI SPDI (36) format.

As a consequence of deep sequencing projects such as gnomAD, we now observe multiple alleles for increasing numbers of short variants. We have refined how the clinical significance assigned by ClinVar (37) is reported in VEP and now match on the input allele rather than just a location. This is important for variants such as rs202155613 (located in BRCA2), where the ‘T’ allele is reported as pathogenic and causes a premature stop gain while the ‘G’ allele is reported as having an uncertain effect or benign.

### Supporting transcript provenance

Clear transcript provenance is a key component of ensuring reliable and reproducible downstream analysis. As new evidence and data sets improve transcript annotation, new transcript records and revisions are continually created. Combined with the availability of multiple transcripts for certain genes, this can make transcript comparison between Ensembl releases a complex and time-consuming process and is especially true for those without bioinformatics support. Ensembl Tark (https://betatark.ensembl.org) records pertinent features of a transcript (e.g. sequence, splicing, assigned symbols) across multiple annotation releases and sources to enable transcript comparison through its web interface and RESTful API. Our beta deployment currently hosts 947 336 transcript records from Ensembl and RefSeq across GRCh37 and GRCh38 and is currently undergoing user testing.

## VISUALIZATIONS FOR GENOMIC ANNOTATION

### Visualizing variation within a 3D context

**Figure 2.**
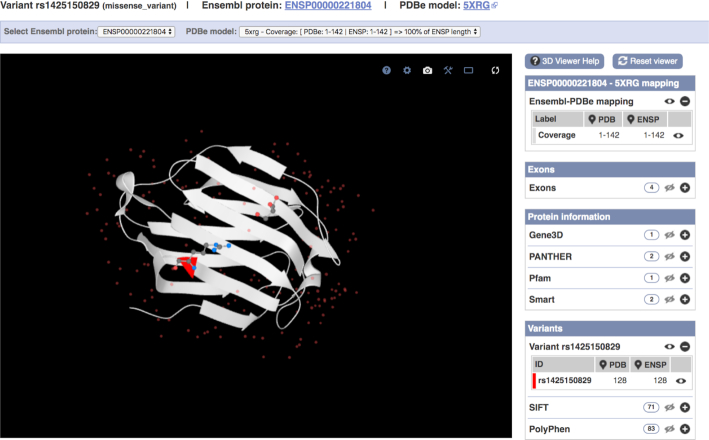
The PDB model 5XRG (linked to ENSP00000221804) is displayed using LiteMol as a Richardson diagram in the central panel. rs1425150829 has been flagged in red at position 128 (ARG) occupying the end of a β strand and shows proximity to a ligand in the 3D structure, suggesting possible disruption. Additional annotation such as exons, protein domains and other variants can be turned on and off by clicking on the associated eye icon on the right hand-side of the visualization.

Variation consequence tools, such as Ensembl VEP, are now an essential step when deciphering a variant's functional impact. Knowing the 3D position of a modified amino acid within a folded protein can also help to understand a variant's impact. Working with the Protein Data Bank in Europe (PDBe) team (38), we have modified the LiteMol viewer (39) to display variant locations on experimentally derived 3D protein structures and have embedded this view into our website and Ensembl VEP tool as seen in Figure 2. UniProt and the Structure Integration with Function, Taxonomy and Sequence (SIFTS) resource (40) provides access to parsimonious observed structures, which can be used to visualize variants in this display. Variants co-located with a transcript can be displayed and coloured according to their SIFT (41) or PolyPhen-2 (42) predicted pathogenicity alongside exon boundaries and protein domains from Gene3D (43), Smart (44), Pfam (45) and Panther (46).

### Working towards a new genome browser

A major focus of the past year has been an extensive redesign and reimplementation of our web interface and infrastructure (a demonstration version is available at: http://2020.ensembl.org). Our new website is developed as a client-side application using the ReactJS framework with data delivered over a set of RESTful web services. Genome visualization is handled by a new tool written in the Rust programming language and transpiled to WebAssembly, which utilises the WebGL API to provide responsive and scalable rendering of genomes from the base pair to chromosome level in a matter of seconds.

For our new site, we are conducting extensive use-case analysis alongside a design driven approach and UX methodologies including stakeholder and user interviews, card sorting exercises (https://www.nngroup.com/articles/usability-testing-1995-sun-microsystems-website/) and usability testing (47). Our aim is to ensure these new interfaces are clear, consistent and usable. Continuous engagement with our growing community is key to ensuring our new interfaces will be fit for purpose. During the third quarter of 2019, we conducted an extensive survey to help prioritise data display on our gene, transcript and variation views. The results of which are being fed into our new gene summary views. We have also conducted a number of one on one interviews with existing users to elucidate common workflows. Interested individuals can sign-up to a Slack workspace where our team is available to discuss any part of the new infrastructure or can subscribe to our mailing lists should they want to participate in user experience sessions. Sign up is available by emailing our helpdesk (helpdesk@ensembl.org).

## TRAINING AND SUPPORT

Ensembl offers training (http://training.ensembl.org/) to researchers across the world where we deliver one of our three courses: an Ensembl browser course aimed at wet-lab researchers and clinicians, an Ensembl REST API course aimed at bioinformaticians and Ensembl Train the Trainer (TtT) courses. Ensembl TtT gives participants resources and skills to teach an Ensembl browser course of their own. All courses can be tailored to suit the needs of a host institute or to fit in as part of a series. We do not charge fees for academic hosts but ask those based in high income countries to support our trainers’ travel and accommodation. Recently, we have released a program to support training in low-middle income countries (https://wellcome.ac.uk/funding/guidance/low-and-middle-income-countries), without those hosts having to bear the additional costs of supporting our officers.

## FUTURE PLANS

As we enter our twentieth year, we see it as one of the most formative of the project's life where we will continue to provide expansive and comprehensive genome annotation, tools to enable genome interpretation and enables researchers to analyse genome data. We plan to annotate and distribute all 260 ordinal level species from VGP once assembled alongside support of DToL. Our release procedures will be revised to accelerate access to genome annotation. Development of our new website will continue as we aim to deliver a minimal viable product during 2020 composed of a genome browser, search, sequence visualization and a custom download tool similar in functionality to BioMart. We will continue to expand MANE Select in collaboration with NCBI to provide as comprehensive coverage of protein coding genes as possible. MANE Select will also replace the Ensembl canonical transcript (a single representative transcript for a locus) where available. Finally, our efforts to annotate regulatory features will expand to fish and cattle as part of our participation in AQUA-FAANG, Gene-SWiTCH and BovReg.

## DATA AVAILABILITY

All Ensembl generated data are available without restriction from our website (https://www.ensembl.org) alongside tools and documentation, in bulk from our FTP site (ftp.ensembl.org) or programmatically via our REST API (https://rest.ensembl.org). Ensembl code is available from GitHub (https://github.com/Ensembl) under an open source Apache 2.0 license. Queries about using Ensembl should be directed to our helpdesk (https://www.ensembl.org/Help/Contact) or to our developer mailing list (https://lists.ensembl.org/mailman/listinfo/dev). Announcements about our services and releases can be found on our blog (https://www.ensembl.info), low-traffic announce mailing list (https://lists.ensembl.org/mailman/listinfo/announce), Twitter (@ensembl) or Facebook (https://facebook.com/Ensembl.org).
